# SCExecute: custom cell barcode-stratified analyses of scRNA-seq data

**DOI:** 10.1093/bioinformatics/btac768

**Published:** 2022-11-30

**Authors:** Nathan Edwards, Christian Dillard, N M Prashant, Liu Hongyu, Mia Yang, Evgenia Ulianova, Anelia Horvath

**Affiliations:** Department of Biochemistry and Molecular & Cellular Biology, Georgetown University, Washington, DC 20057, USA; Department of Biochemistry and Molecular Medicine, McCormick Genomics and Proteomics Center, School of Medicine and Health Sciences, The George Washington University, Washington, DC 20037, USA; Department of Biochemistry and Molecular Medicine, McCormick Genomics and Proteomics Center, School of Medicine and Health Sciences, The George Washington University, Washington, DC 20037, USA; Departments of Genetics and Genomic Sciences, Icahn School of Medicine at Mount Sinai, New York, NY 10029, USA; Department of Biochemistry and Molecular Medicine, McCormick Genomics and Proteomics Center, School of Medicine and Health Sciences, The George Washington University, Washington, DC 20037, USA; Division of Animal Sciences, University of Missouri, Columbia, MO 65211, USA; Department of Biochemistry and Molecular Medicine, McCormick Genomics and Proteomics Center, School of Medicine and Health Sciences, The George Washington University, Washington, DC 20037, USA; Department of Biochemistry and Molecular Medicine, McCormick Genomics and Proteomics Center, School of Medicine and Health Sciences, The George Washington University, Washington, DC 20037, USA; Department of Biochemistry and Molecular Medicine, McCormick Genomics and Proteomics Center, School of Medicine and Health Sciences, The George Washington University, Washington, DC 20037, USA; Department of Biochemistry and Molecular Medicine, School of Medicine and Health Sciences, The George Washington University, Washington, DC 20037, USA

## Abstract

**Motivation:**

In single-cell RNA-sequencing (scRNA-seq) data, stratification of sequencing reads by cellular barcode is necessary to study cell-specific features. However, apart from gene expression, the analyses of cell-specific features are not sufficiently supported by available tools designed for high-throughput sequencing data.

**Results:**

We introduce SCExecute, which executes a user-provided command on barcode-stratified, extracted on-the-fly, single-cell binary alignment map (scBAM) files. SCExecute extracts the alignments with each cell barcode from aligned, pooled single-cell sequencing data. Simple commands, monolithic programs, multi-command shell scripts or complex shell-based pipelines are then executed on each scBAM file. scBAM files can be restricted to specific barcodes and/or genomic regions of interest. We demonstrate SCExecute with two popular variant callers—GATK and Strelka2—executed in shell-scripts together with commands for BAM file manipulation and variant filtering, to detect single-cell-specific expressed single nucleotide variants from droplet scRNA-seq data (10X Genomics Chromium System).

In conclusion, SCExecute facilitates custom cell-level analyses on barcoded scRNA-seq data using currently available tools and provides an effective solution for studying low (cellular) frequency transcriptome features.

**Availability and implementation:**

SCExecute is implemented in Python3 using the Pysam package and distributed for Linux, MacOS and Python environments from https://horvathlab.github.io/NGS/SCExecute.

**Supplementary information:**

Supplementary data are available at *Bioinformatics* online.

## 1 Introduction

In single-cell RNA-sequencing (scRNA-seq) data cell barcodes are used to extract cell-specific sequencing reads and assess cell-level features. Methods for cell-specific scRNA-seq analysis have been focused on gene expression, where popular approaches—such as STARsolo and CellRanger—integrate alignment with concurrent barcode demultiplexing and the assignment of read counts to genes (Kaminow *et al.*, 2021; Tran *et al.*, 2019). Additional cell-level transcriptome feature analyses—for example, expressed genetic variation, allele-specific expression and splicing—are now beginning to emerge, demonstrating the substantial information content of scRNA-seq data (La Manno *et al.*, 2018; Larsson *et al.*, 2021; Liu *et al.*, 2021; Prashant *et al.*, 2020, 2021a, b; Schnepp *et al.*, 2019; Vu *et al.*, 2019). These types of analyses can benefit from widely applicable cell-level read-stratifying methods.

To facilitate custom cell-level analyses of scRNA-seq data, we have developed SCExecute, software that manages the execution of a desired command on barcode-stratified single-cell binary alignment map (scBAM) files. SCExecute generates scBAMs on-the-fly by extracting barcodes from the QNAME field or tags of barcoded, aggregated single-cell alignments, produced by widely used scRNA-seq tools, such as CellRanger, STARsolo and UMI-tools (Smith *et al.*, 2017). scBAMs and the respective downstream analyses can be restricted to a user-specified list of barcodes, such as the filtered list of cell barcodes generated by STARsolo, or user-selected barcodes of interest. The user-specified command option executes in a sub-shell, suitable for simple commands, monolithic programs, multi-command shell scripts and complex shell-based pipelines. ScBAMs can optionally be filtered for reads aligned to genomic regions of interest.

## 2 Software description

### 2.1 Implementation

SCExecute is modeled after the classic Linux tools *find* and *xargs*, which execute a command repeatedly on a collection of files. Instead of generating scBAMs for all cellular barcodes first, and only then executing a command on each scBAM, SCExecute interleaves the generation of a batch of scBAMs and execution of the command—avoiding operating system limits, such as the number of open file handles and available memory, while enabling maximum throughput on multiprocessor systems. Users can specify the number of commands to run at once and adjust the batch size to ensure that all processors are kept busy. User commands are executed in the same context as the user’s command-line shell—so simple commands, monolithic programs, multi-command shell scripts and complex commands including pipes, list operators and standard input, output and error redirection work as usual.

SCExecute permits a flexible and user-configurable approach to extracting cell barcodes from aggregated scRNA-seq BAM files generated by various scRNA-seq-processing tools, which employ different read-tags and/or read-naming strategies. Explicit configuration is provided for alignments barcoded using CellRanger, STARsolo and UMItools. Users can also configure novel cellular barcode extraction logic based on BAM file tags, or values in the QNAME field with delimited tokens or regular expressions. The generated scBAM filename and other execution-specific parameters are inserted into the command before execution using the same syntax as *find* and *xargs* for the location of the generated filename (‘{}’). In addition, the cell-barcode is available for substitution using ‘{BARCODE}’ and the name of the input BAM file containing pooled scRNA-Seq alignments, without its path or the ‘.bam’ extension, is available as ‘{BAMBASE}’. If there are no replacement tokens in the command template, the generated scBAM filename is added at the end of the command. Users can also specify templates for generated scBAM filenames, working directories and filenames for standard output and/or standard error. Each SCExecute pass through the BAM file extracts scBAMs for a user-configurable number of cell barcodes. Usually extracting 100–200 scBAMs per pass is sufficient to keep the command execution CPUs busy. The generated scBAMs retain the header fields and sorted order of the BAM file provided as input. An option is also provided to index the scBAMs using *samtools* prior to executing the user-supplied command. SCExecute manages the creation, indexing, analysis of, and deletion of scBAMs.

For focused analyses or debugging, SCExecute can be restricted to specific genomic regions and can limit the number of generated scBAMs. SCExecute can be configured to use cleaned-up cell barcodes (e.g. STARsolo CB tags), raw cell barcodes (e.g. STARsolo CR tags), to use a list of acceptable cell barcodes (e.g. STARsolo barcodes.tsv output) or all cell-barcodes found in the BAM file.

SCExecute is freely available as a self-contained binary package for 64-bit Intel-based Linux and MacOS systems, as Python3 source, and as multi-platform *Bioconda* package (https://horvathlab.github.io/NGS/SCExecute).

### 2.2 Example of use

**Fig. 1.**
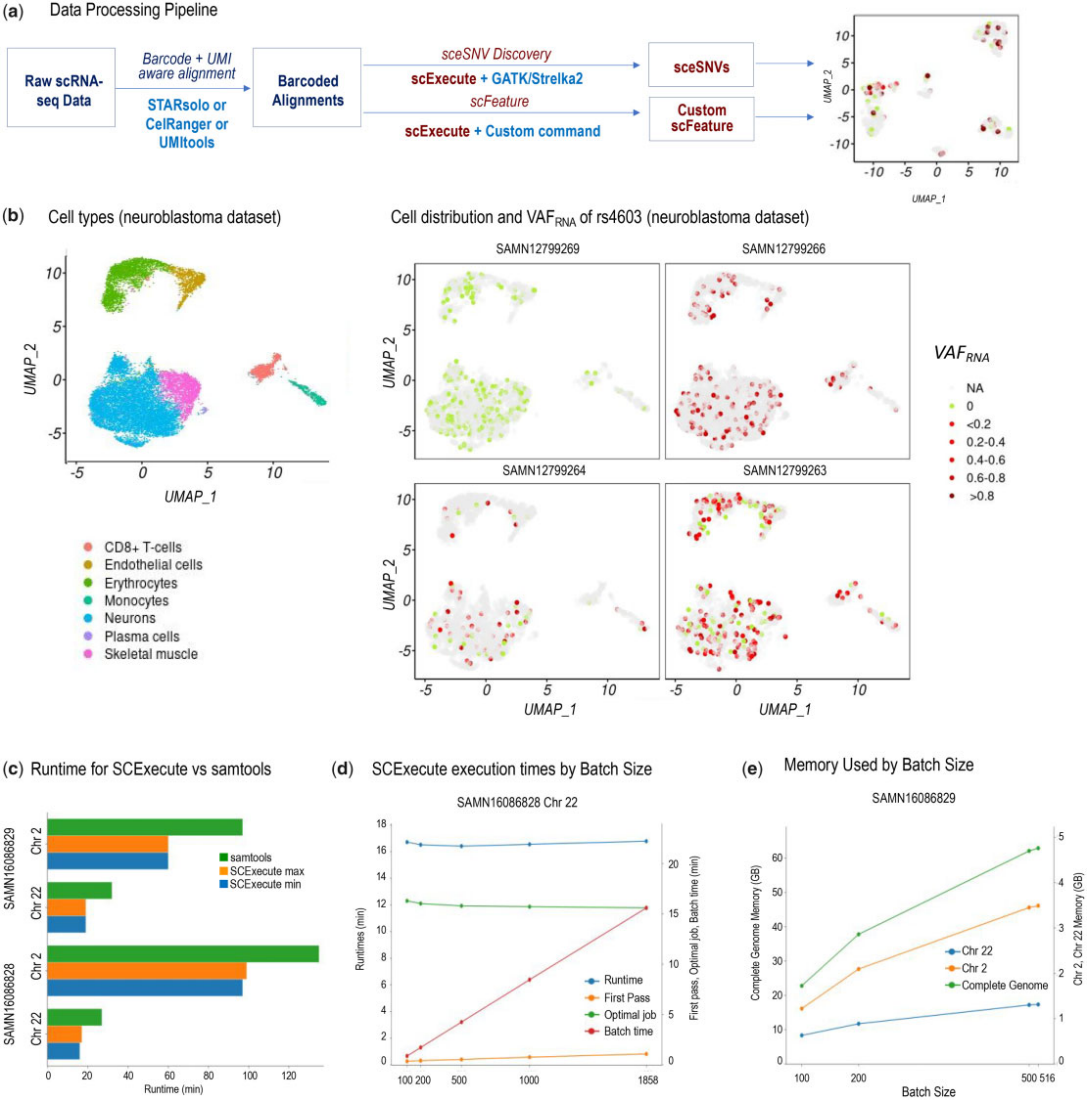
(**a**) SCExecute data processing. (**b**) UMAP projections showing neuroblastoma dataset cells classified by type (left), and cell distribution and cellular expressed variant allele frequency (VAF_RNA_) of the missense substitution rs4603 (1:151401549_T > C) in the gene *PSMB4* (right). Cells in which the SNV locus is covered by less than 5 reads and the VAF_RNA_ is not accessible are indicated as “NA”. VAF_RNA_=0 indicates cell where all the reads (5 and more) covering the SNV locus carried the reference nucleotide (See Supplementary Methods). In cells where at least 5 reads cover the SNV locus and 1>VAF_RNA_>0 the color intensity shows the relative expression of the sceSNV. The rs4603 VAF_RNA_ cell distribution is consistent with germline homozygous variant in sample SAMN12799266, heterozygous variant in samples SAMN12799264 and SAMN12799263, and absence (homozygous reference) in sample SAMN12799269. (**c**) SCExecute runtimes in comparison with related *samtools* function for Chr 2 and Chr 22 of samples SAMN16086828 and SAMN16086829. The *samtools-*based approach, which extracts each barcode’s alignments one at a time independently and in parallel, requires more runtime than the SCExecute approaches. (**d**) SCExecute execution times by batch size. The time to construct the first batch size cell-specific scBAMs (First pass) is approximately constant for all Batch Size values. (**e**) SCExecute memory use by batch size. The memory footprint increases with batch size to accommodate the reads of the scBAM files for the current batch

We demonstrate SCExecute using two variant callers—GATK and Strelka2—run from a shell script with additional utilities for BAM handling and variant filtering (*samtools*, *bcftools*) (Kim *et al.*, 2018; Li *et al.*, 2009; Van der Auwera *et al.*, 2013). We used 10 publicly available scRNA-seq datasets generated on the 10xGenomics Chromium-3’UTR system from a variety of samples, including adrenal neuroblastoma, prostate cancer and MCF7 cells, from three different studies (Dong *et al.*, 2020; Ma *et al.*, 2020; Ben-David *et al.*, 2018). The data processing is shown on Figure 1a (see also Supplementary Methods and Supplementary Figs S1 and S2).

Briefly, pooled scRNA-seq sequencing reads were aligned using the STARsolo module of STAR v.2.7.7a in 2-pass mode, against human genome assembly GRCh38.79 and corresponding transcript annotations. Using SCExecute, variant calling was performed on the aligned reads of each cell, applying the HaplotypeCaller module of GATK v.4.2.0.0 and Strelka2 v.2.9.10 in parallel, followed by BCFtools variant quality filtering. This analysis identified as many as 70K single-cell-specific expressed single nucleotide variants (sceSNVs) in two or more cells per sample (Figure 1b and Supplementary Table S1). From those, between 11% and 36% sceSNVs per dataset were not previously reported in the single nucleotide polymorphism database (dbSNP, Sherry *et al.*, 1999, Supplementary Table S2). For sceSNVs of interest, the corresponding scBAMs, optionally restricted to the sceSNV regions, can be saved using the SCExecute ‘file template’ option, and further explored, for example, through the integrative genomics viewer (Robinson *et al.*, 2011), Supplementary Figure S4.

### 2.3 Performance

We compared the performance of SCExecute and *samtools*-based workflows on different-sized scRNA-Seq BAM files with different numbers of cellular barcodes and reads per barcode. We observed that, first, the *samtools*-based approach, which extracts each barcode’s alignments one at a time independently and in parallel, requires 35–66% more **Runtime** than the SCExecute approaches, even for small **Batch Size** (Fig. 1c and Supplementary Fig. S5). Second, the time to construct the first **Batch Size** cell-specific scBAMs (**First pass**) is approximately constant for all **Batch Size** values (Fig. 1d and Supplementary Fig. S6). The **Optimal Job** time estimates the time to execute the jobs on eight CPUs with no overhead, based on the average job execution time. For the second and subsequent passes through the BAM file, the BAM File I/O thread is competing for CPU time with job execution, so the **Optimal Job** time is reduced slightly with increasing **Batch Size**. Third, the memory footprint increases with **Batch Size** to accommodate the reads of the current batch (Fig. 1e and Supplementary Fig. S7). It is feasible, for some pooled scRNA-Seq BAMs, to use such a large **Batch Size** value that only the first pass is needed if sufficient memory is available. If the **Batch time** is larger than the **First pass** time (as shown in these examples), the workers will not finish execution of each batch’s scBAMs before the next pass through the pooled scRNA-Seq file has completed, and the workers will never wait for more jobs after the first pass is done. Once the **Batch time** is longer than the **First pass** time, the total running time of SCExecute is essentially constant.

As this analysis demonstrates, the running time of SCExecute-based barcode-stratified analyses is driven largely by the execution time of the user-supplied command applied to each scBAM file. The primary execute-time attributable to SCExecute itself is the construction of the first batch of scBAMs—subsequent passes through the pooled scRNA-Seq BAM file are carried out in parallel with the execution of the user-supplied command and only as needed. For large enough batch sizes, applying the user-supplied command to each scBAM of the batch will take longer than each SCExecute pass through the pooled scRNA-Seq data. The SCExecute running time to create a batch of scBAMs is essentially invariant to the size of the batch and involves primarily I/O to access each alignment, extract the cellular barcode, and write out (some of) the reads. SCExecute will optionally use multiple processors to execute the user-supplied command in parallel. Total running time of a SCExecute-based analysis will therefore depend on the number of cellular barcodes, the execution time of the user-specified command, the number of processors used, and the time to create the first batch of scBAM files. Unless the user-specified command is very quick, the SCExecute-specific contribution to running time is modest.

For the genome-wide GATK variant call script applied to cellular barcode-stratified scBAMs, scRNA-Seq dataset SAMN09210328 contains 1858 cell-barcodes after alignment using STARsolo and filtering (Supplementary Table S3). The GATK variant calls script required, on average, about 3 min per scBAM, resulting in a total (wall-clock) execution time of about 726 min on eight processors and a SCExecute running-time overhead of less than 5%.

## 3 Discussion

Cell-level transcriptome analyses are essential to understand the details of each cell’s expressed features. To aid these analyses, we have developed SCExecute, which provides an effective solution for custom cell-specific analyses from scRNA-seq data using existing tools, especially those designed for bulk RNA- and DNA-sequencing data.

We demonstrate SCExecute with variant callers designed for bulk (DNA-)sequencing data to identify sceSNVs. This analysis identified over 10 000 high-quality non-dbSNP SNVs across 10 datasets and 51 411 cells. SceSNVs from 10xGenomics scRNA-seq data are vastly understudied, as traditional variant callers estimate quality metrics, including allele frequency and/or genotype confidence, based on all reads. As a result, SNVs with low allele frequency and/or uncertain genotypes in pooled scRNA-Seq data are discarded. At the same time, it is well acknowledged that post-zygotic SNVs (such as somatic and mosaic mutations), being present in only a proportion of cells, can result in low allele frequency. Similar considerations apply to RNA-source variations, such as those resulting from RNA-editing or transcriptional infidelity. Importantly, analogous issues are expected for all cell-specific features, including splicing and allele-specific expression. Cell-specific scBAM analyses managed by SCExecute help expose unique and specific cellular features and discover low (cellular) frequency transcriptome features. Finally, SCExecute can readily be integrated with workflow managers and can provide a viable solution that avoids the added complexity of a general workflow manager.

## Supplementary Material

btac768_Supplementary_Data
