# Supplementary material for: SCExecute: custom cell barcode-stratified analyses of scRNA-seq data
Source: Bioinformatics. 2022 Nov 30;39(1):btac768. doi: 10.1093/bioinformatics/btac768 (PMC9825775; doi:10.1093/bioinformatics/btac768)
Supplement: btac768_Supplementary_Data [file btac768_supplementary_data.zip › scExecute_suppinfo_111122_f.pdf]

SCExecute: cell barcode-stratified analyses of scRNA-seq data

Nathan Edwards[1#], Christian Dillard[2], Prashant NM[2,3], Hongyu Liu[2,4], Mia Yang[2], Evgenia Ulianova[2], and Anelia Horvath[2,6#]

#Correspondence

[1]Department of Biochemistry and Molecular & Cellular Biology, Georgetown University, Washington, DC 20057, USA
[2]McCormick Genomics and Proteomics Center, School of Medicine and Health Sciences, The George Washington University, Washington, DC, 20037, USA
[3] Departments of Genetics and Genomic Sciences, Icahn School of Medicine at Mount Sinai, New York, NY 10029, USA
[4]Division of Animal Sciences, University of Missouri, Columbia, MO, 65211, USA
[5]Department of Biochemistry and Molecular Medicine, School of Medicine and Health Sciences, The George Washington University, Washington, DC, 20037, USA

Supplementary Information
(in the order of appearance in the main text)

Supplementary Methods with References		p.2
Figure_S1.	Features and readconts distribution of the used datasets	p.4
Figure_S2.	Heatmaps showing cell-types similarity to known cell types (SingleR)	p.5
Figure_S3.	Distribution and expression of the sceSNV COSV56936745 in prostate sets	p.6
Figure_S4.	IGV visualization of sceSNV-positive cells	p.7
Table_S1.	SceSNVs statistics and technical metrics.	p.8
Table_S2.	*Submitted as a separate file*	
SCExecute performance tests		p.9
Table_S3.	Datasets included in the performance tests	p.10
Figure_S5.	SCExecute vs samtools Runtimes	p.11
Figure_S6.	SCExecute Execution Times by Batch Size	p.12
Figure_S7.	SCExecute Memory Footprint by Batch Size	p.14
Discussion of SCExecute performance		p.15

Supplementary Methods

Sequencing Datasets

To exemplify SCExecute we utilized publicly available scRNA-seq data from three different cancer studies providing prostate cancer tissue (Ma *et al.*, 2020), neuroblastoma (Dong *et al.*, 2020) and the human breast carcinoma cell line MCF7 (Ben-David *et al.*, 2018). The experimental design and the data generation are described in detail in the original studies. All the studies utilized 10x Genomics Chromium Single Cell 3′ Workflow protocol for the libraries' generation, and the libraries were sequenced on an Illumina NextSeq 500 platform. The sequencing datasets were downloaded from the NCBI Sequence Read Archive (SRA) (accessed on 1 April 2021) under the accession numbers listed in Table_S1.

Alignment and Variant call

The pooled raw scRNA-seq sequencing reads were aligned using the STARsolo module of STAR v.2.7.7a in 2-pass mode, with transcript annotations assembly GRCh38.79 (Kaminow *et al.*, 2021). Transcriptome-wide variant calling was performed on each SCbam using SCExecute in conjunction with the HaplotypeCaller module of GATK v.4.2.0.0 and Strelka2 v.2.9.10 in parallel; both tools were used in their default setting (Van der Auwera *et al.*, 2013; Kim *et al.*, 2018). The GATK HaplotypeCaller was preceded by the assignment of read groups using the GATK module AddOrReplaceReadGroups, followed by splitting reads that contain Ns in their cigar string with the GATK module SplitNCigarReads.

For our analysis, we focused on the SNVs (i.e. indels and other variants were filtered out). The sceSNV calls from the individual alignments were filtered using the bcftools utility (v.1.10.2) of SAMtools retaining sceSNVs with QUAL (Phred-scaled probability) > 100, MQ (mapping quality) > 60, and QD (quality by depth) > 2. SNV loci were annotated using SeattleSeq v.16.00 (dbSNP build 154), and SNVs positioned in non-repetitive regions were retained for further analysis. For sceSNVs of interest, the corresponding SCbams, optionally restricted to the sceSNV regions, can be saved using the SCExecute "file template" option, and further explored, for example, through the Integrative Genomics Viewer (IGV, (Robinson *et al.*, 2011), Figure 1c.

Quality Assessments

To define likely cell types, we used read-count matrices with the raw gene counts per cell generated by STARsolo. We normalized and scaled the expression data using the SCTransform function, as implemented in Seurat v.3.0 (Butler *et al.*, 2018). The cell-feature distributions were then plotted to identify and filter out the outliers and low-quality cells, which we defined after examination of the cell feature distribution (Supplementary Figure_S1). Specifically, based on the cell and feature distribution, we have filtered out: (1) cells with mitochondrial gene expression of above between 10% and 20%, (2) cells with fewer than 1000 genes, and (3) cells with more than between 3500 and 5500 detected genes (to remove potential doublets). The Seurat-processed gene expression values were also used to remove batch effects and cell cycle effects, as well as for cell type assessments.

Cell Types Classifications

To define likely cell types with known cell types, we used SingleR v.1.0.5 (Aran *et al.*, 2019), as previously described (Liu *et al.*, 2021; N. Prashant *et al.*, 2021; N. M. Prashant *et al.*, 2021). SingleR defines likely cell types, comparing genome-wide expression profile of each cell to a database of reference cells' whole transcriptome expression (BluePrint + ENCODE datasets). To select the expression profile corresponding to known cell type, the analysis is rerun iteratively with the top cell types from the previous step.

VAF_{RNA} Estimation and SNV Distribution Plotting

Single-cell level VAFRNA was assessed from the pooled scRNA-seq alignments using scReadCounts v.1.1.4, as we have previously described (N. M. Prashant et al., 2021). When provided with barcoded scRNA-

seq alignments and genomic loci of interest (with alleles), SCReadCounts tabulates the reference and variant read counts (n_{ref} and n_{var}, respectively), and generates a cell-SNV matrix with the VAF_{RNA} estimated at a user-defined threshold of the minimum number of required sequencing reads (minR) for a confident VAF_{RNA} assessment. For the analysis presented herein, we used minR ≥ 5, which excludes from the estimation those positions covered by an insufficient number of reads (in this case 5). The cell-SNV VAF_{RNA} matrix is than used as an input together with outputs of Seurat and SingleR to plot the SNV-distributions on the two-dimensional UMAP projections.

References

Aran,D. *et al.* (2019) Reference-based analysis of lung single-cell sequencing reveals a transitional profibrotic macrophage. *Nat. Immunol.*

Van der Auwera,G.A. *et al.* (2013) From fastQ data to high-confidence variant calls: The genome analysis toolkit best practices pipeline. *Curr. Protoc. Bioinforma.*

Ben-David,U. *et al.* (2018) Genetic and transcriptional evolution alters cancer cell line drug response. *Nature.*

Butler,A. *et al.* (2018) Integrating single-cell transcriptomic data across different conditions, technologies, and species. *Nat. Biotechnol.*

Dong,R. *et al.* (2020) Single-Cell Characterization of Malignant Phenotypes and Developmental Trajectories of Adrenal Neuroblastoma. *Cancer Cell.*

Kaminow,B. *et al.* (2021) STARsolo: accurate, fast and versatile mapping/quantification of single-cell and single-nucleus RNA-seq data. *bioRxiv.*

Kim,S. *et al.* (2018) Strelka2: fast and accurate calling of germline and somatic variants. *Nat. Methods.*

Liu,H. *et al.* (2021) scReQTL: an approach to correlate SNVs to gene expression from individual scRNA-seq datasets. *BMC Genomics*, **22**, 40.

Ma,X. *et al.* (2020) Identification of a distinct luminal subgroup diagnosing and stratifying early stage prostate cancer by tissue-based single-cell RNA sequencing. *Mol. Cancer.*

Prashant,N. *et al.* (2021) Improved SNV discovery from barcode-stratified scRNA-seq alignments. *Genes (Basel).*, **12**.

Prashant,N.M. *et al.* (2021) SCReadCounts: estimation of cell-level SNVs expression from scRNA-seq data. *BMC Genomics*, **22**, 689.

Robinson,J.T. *et al.* (2011) Integrative genomics viewer. *Nat. Biotechnol.*, **29**, 24–26.

Figure_S1. Features (genes) and readconts distribution for the prostate cancer dataset (a) and the neuroblastoma dataset (b).

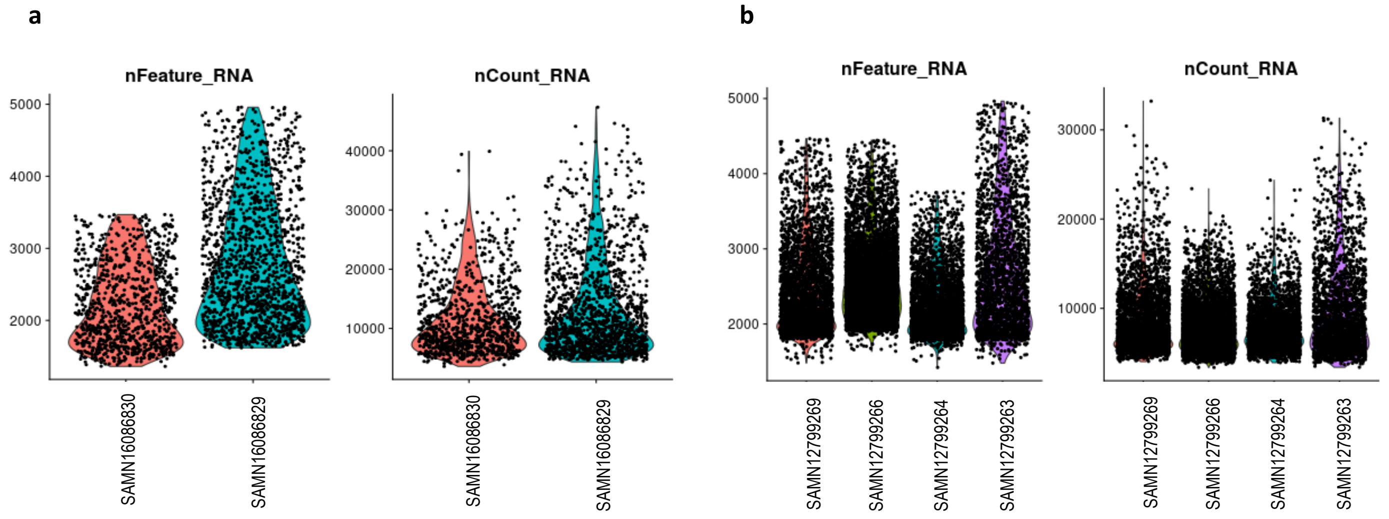

Figure_S2. Heatmaps showing cell-types similarity to known cell types of the sample SAMN16086829 from the prostate cancer dataset (a) and samples SAMN12799269 from the neuroblastoma dataset as determined by SingleR.

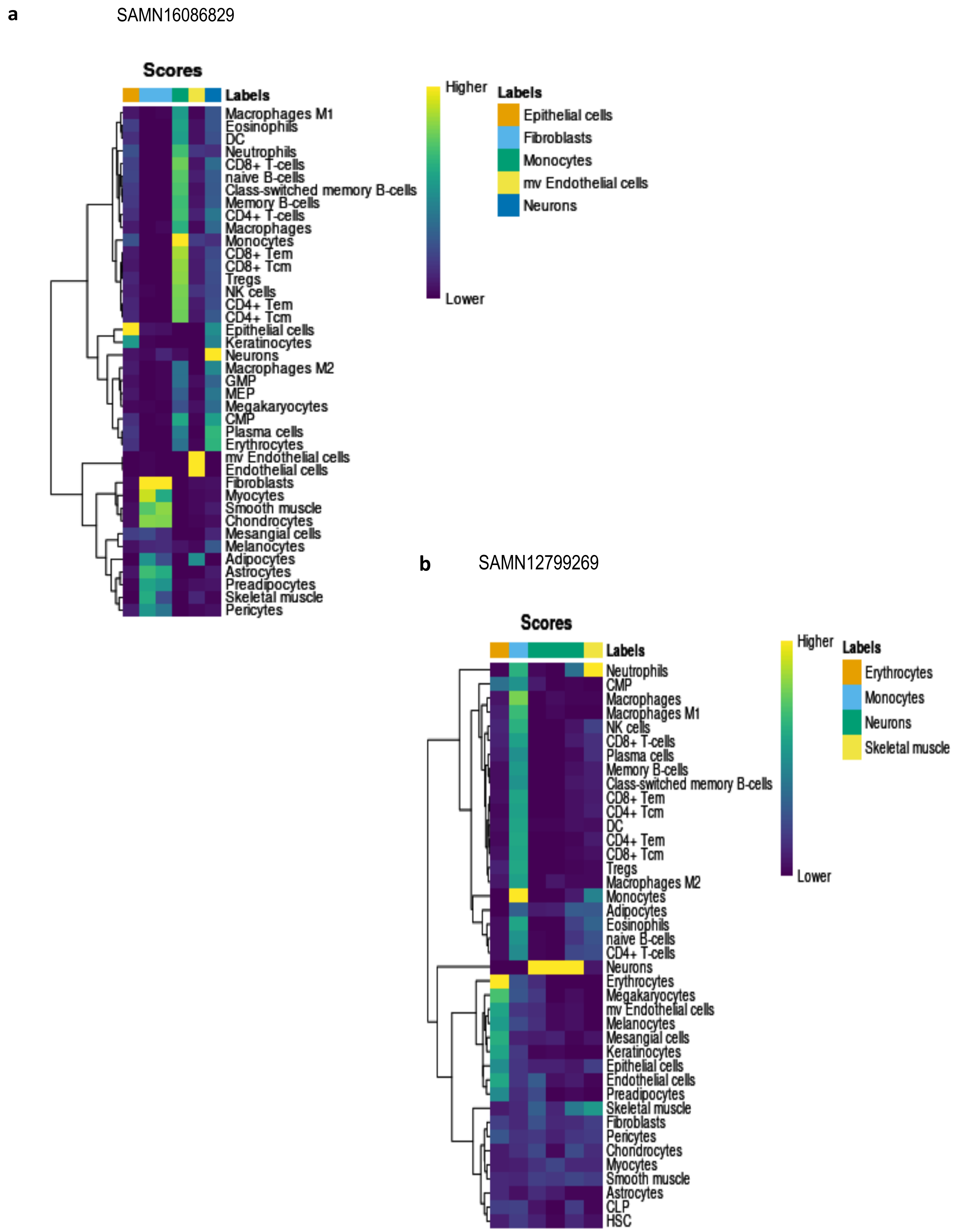

Figure_S3. UMAP projections showing cells classified by type (a) and the cell distribution and VAF_{RNA} of the missense substitution rs1051447 (also reported as a somatic mutation, COSV56936745) in the gene *CWH43* in the two prostate cancer samples (b). The *CWH43* and COSV56936745 are mostly expressed in neurons.

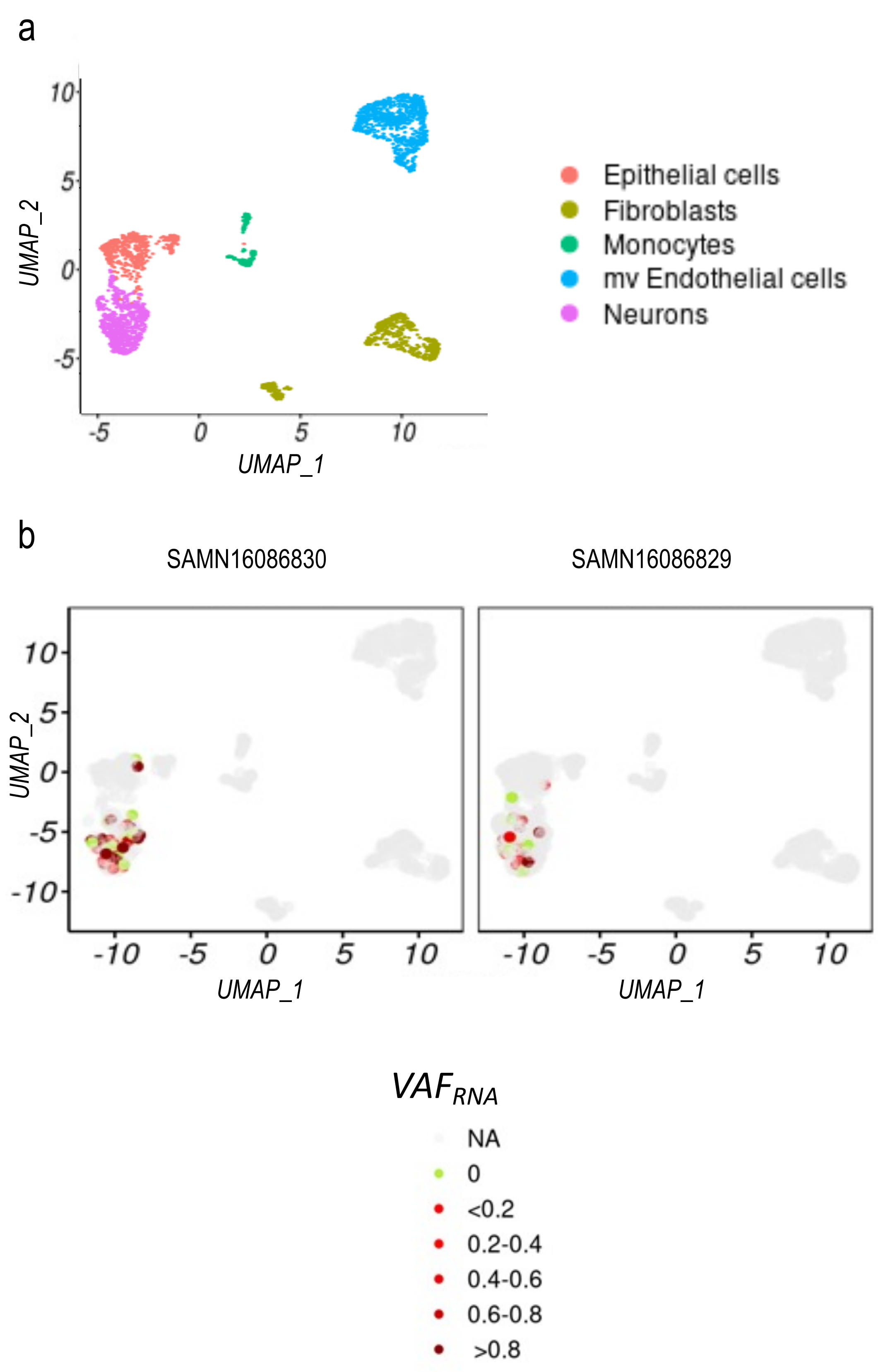

Figure_S4. Integrative Genome Viewer (IGV) visualization of sceSNV-positive cells (COSV56936745) from sample SAMN16086830 shows mono-and bi-allelic expression of the SNV.

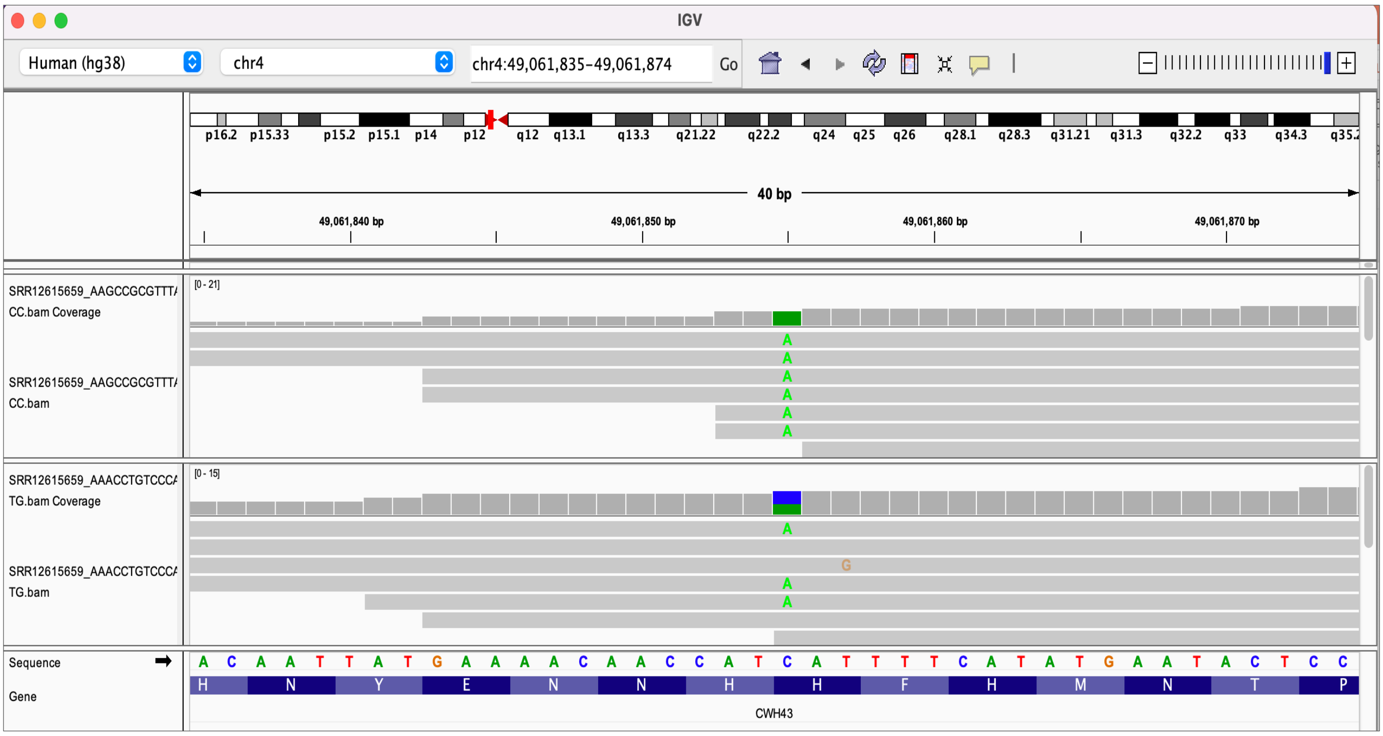

Table_S1. SceSNVs statistics and technical metrics in 10 scRNA-seq sets.

Source	SampleID	seq read length	N cells	Mean Reads/ cell	Mean UMI/ cell	Total N sceSNVs in 2+ cells	non-DbSNP sceSNVs (novel) in 2+ cells	
							Total N	per cell
prostate cancer	SAMN16086830	150	1455	121811	14298	68797	22020	15,1
	SAMN16086829	150	2019	80528	14810	59708	19451	9,6
neuro-blastoma	SAMN12799269	150	6994	20892	6969	53872	19217	2,7
	SAMN12799266	150	12448	13313	5742	45640	12653	1,0
	SAMN12799264	150	16554	9140	4446	36877	9731	0,6
	SAMN12799263	150	4273	28516	8512	44654	15263	3,6
MCF7	SAMN09210331	100	1749	37605	21682	7058	780	0,4
	SAMN09210329	100	2778	20836	15222	3784	528	0,2
	SAMN09210328	100	1891	38597	28034	3432	411	0,2
	SAMN09210327	100	1250	52928	28223	5190	661	0,5
Statistics			sum 51411	mean 41249	mean 13302	sum 329012	sum 100715	mean 3,4

SCExecute performance tests

SCExecute is run on an eight CPU, 64 GB Dell 1U Dell R410 computer running CentOS 7, using the following command (for Chr 2, Batch Size 100, BAM file from SRA sample SAMN16086829 after alignment with STARsolo as described in the manuscript). The command assumes SCExecute, scBAMStats, and samtools are on the current path:

```
% scExecute -r "SAMN16086829_wasp_Aligned.sortedByCoord_vW_filt.bam" \
  -C "echo -n "{BARCODE} reads "; samtools view {} | wc -l; samtools index {}; scBAMStats {}" \
  -O "{BARCODE}.out" -B 100 -R "2" -t 8 -b "barcodes.tsv"
```

This effectively executes the follow commands on each scBAM file:

```
echo -n "{BARCODE} reads "
samtools view {} | wc -l
samtools index {}
scBAMStats {}
```

The scBAMStats tool is distributed with SCExecute, and counts cellular-barcodes, UMIs, and coverage per UMI.

Samtools-based Strategy

We also implemented a samtools-based strategy using samtools view with its “--tag" option to extract only reads with a specific barcode in the CB tag, in conjunction with the STARsolo barcodes.tsv file and the xargs utility to execute on each of the eight CPUs (for Chr 2). On the command-line:

```
% cat barcodes.tsv | \
  xargs -P 8 -i ./commands.sh SAMN16086829_wasp_Aligned.sortedByCoord_vW_filt.bam 2 {}
```

The commands.sh script has the following:

```
#!/bin/sh
ORIGBAM=$1
REGION=$2
BARCODE=$3
BAMFILE=${BARCODE}.bam
( samtools view --tag CB:${BARCODE} ${ORIGBAM} ${REGION} -b -o ${BAMFILE}; \
  echo -n "${BARCODE} reads "; \
  samtools view ${BAMFILE} | wc -l; \
  samtools index ${BAMFILE}; \
  scBAMStats ${BAMFILE}; \
) >${BARCODE}.out 2>&1
rm -f ${BAMFILE} ${BAMFILE}.bai
```

Terminology

The following times are captured and shown in the figures below:

Runtime Total wallclock running time.
First pass Time to extract the first Batch Size cell-specfic scBAM files for execution.
Optimal Job Minimal time to execute all cell-specific jobs using eight workers. Based on the average job execution time for a particular batch size.
Batch Time Expected time to execute the one Batch size batch of cell-specific jobs using eight workers. Based on the average job execution time.

Table_S3. Datasets included in the performance tests

SRA Sample	Restriction	STARsolo Barcodes	Reads	Reads per Barcode Mean	Reads per Barcode Median	Avg. Job Runtime (sec.)
SAMN09210328	Chr 22 Only	1858	1656450	891.52	794	4.21
SAMN09210328	Chr 2 Only	1858	5993021	3225.52	2907	24.52
SAMN09210328	Whole Genome	1858	113524661	61100.46	54668	380.00
SAMN16086829	Chr 22 Only	516	824599	1598.06	909	16.01
SAMN16086829	Chr 2 Only	516	2325736	4507.24	2777	50.99
SAMN16086829	Whole Genome	516	44159469	85580.37	52042	1640.00

Figure_S5. Runtime for SCExecute vs Samtools

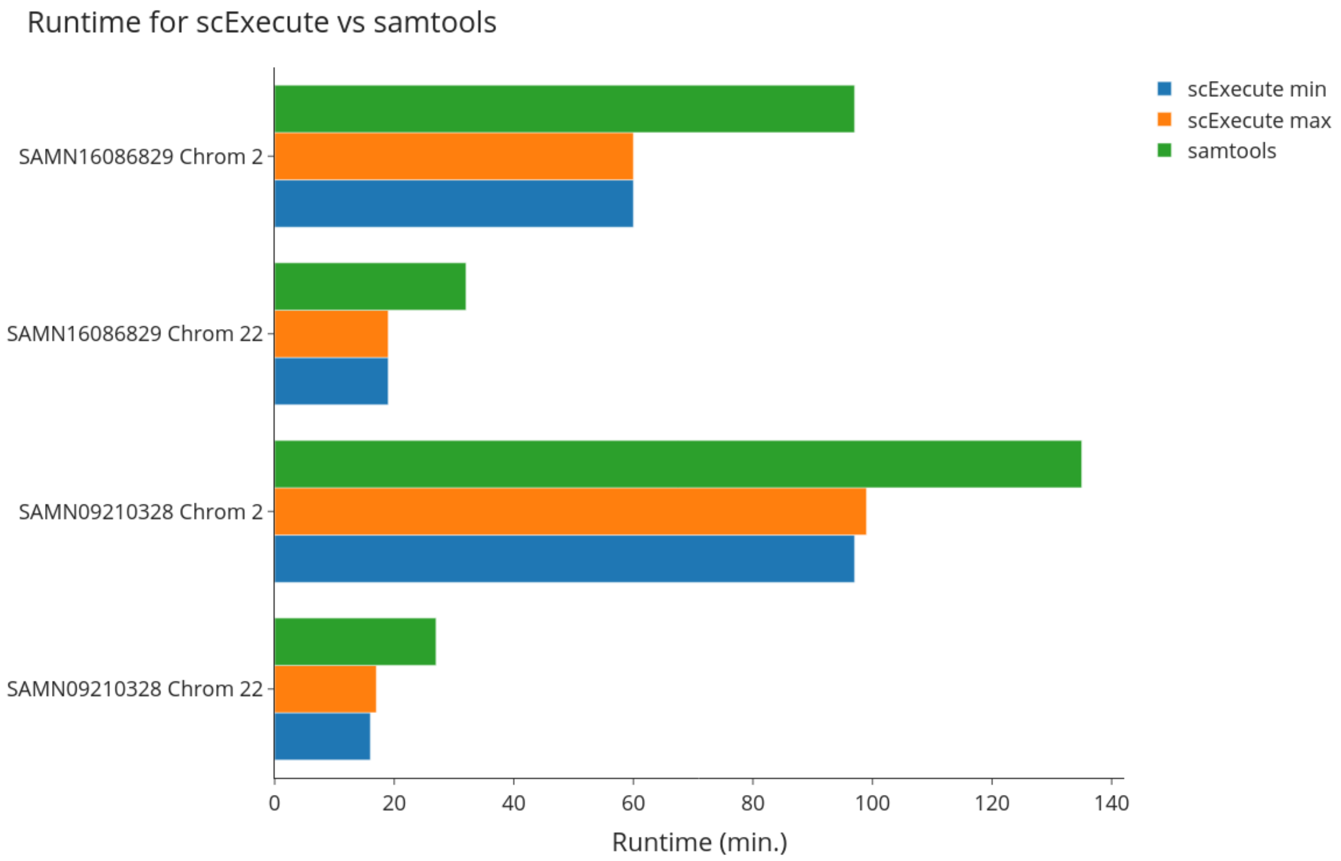

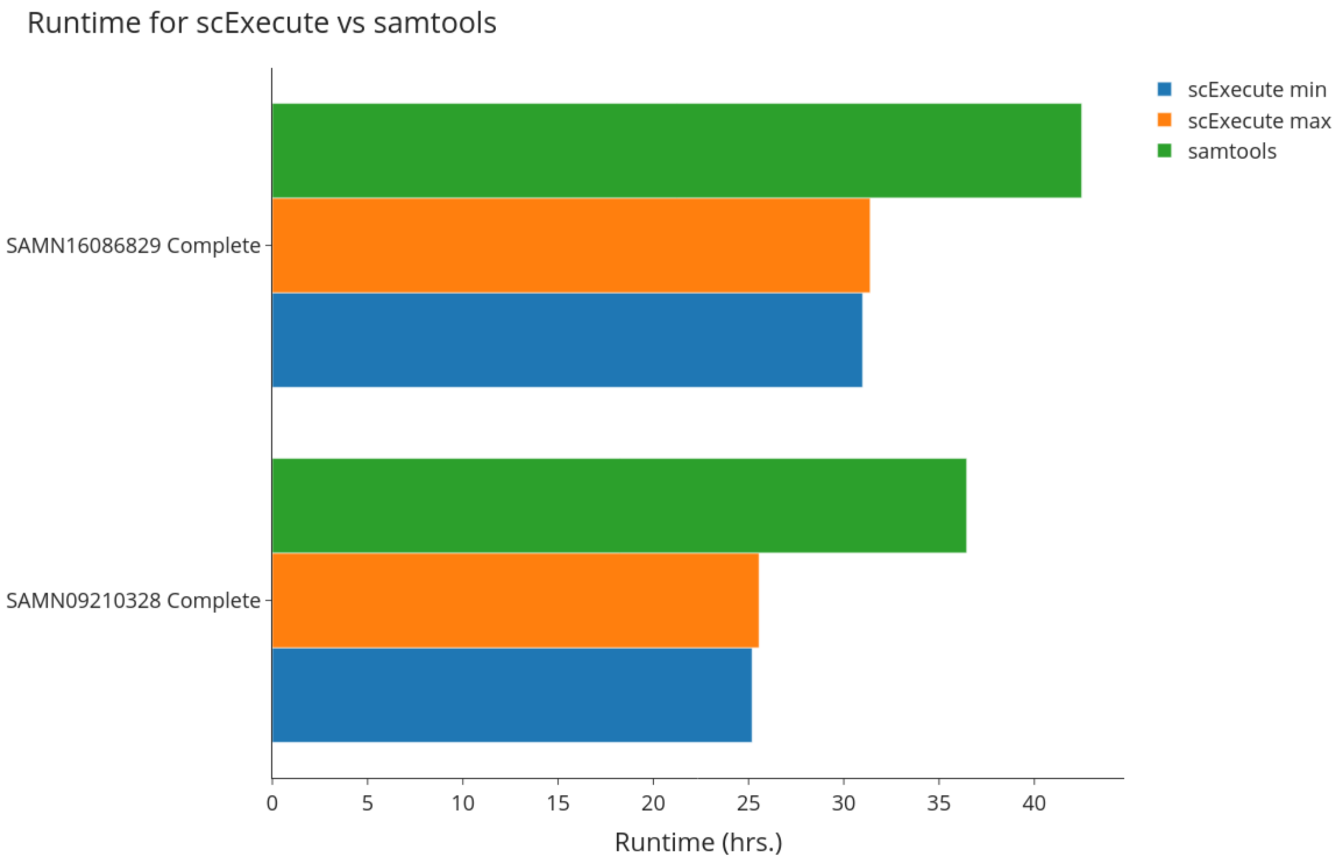

Figure_S6. SCExecute Execution Times by Batch Size for dataset SAMN09210328 (a) and SAMN09210329 (b)

a

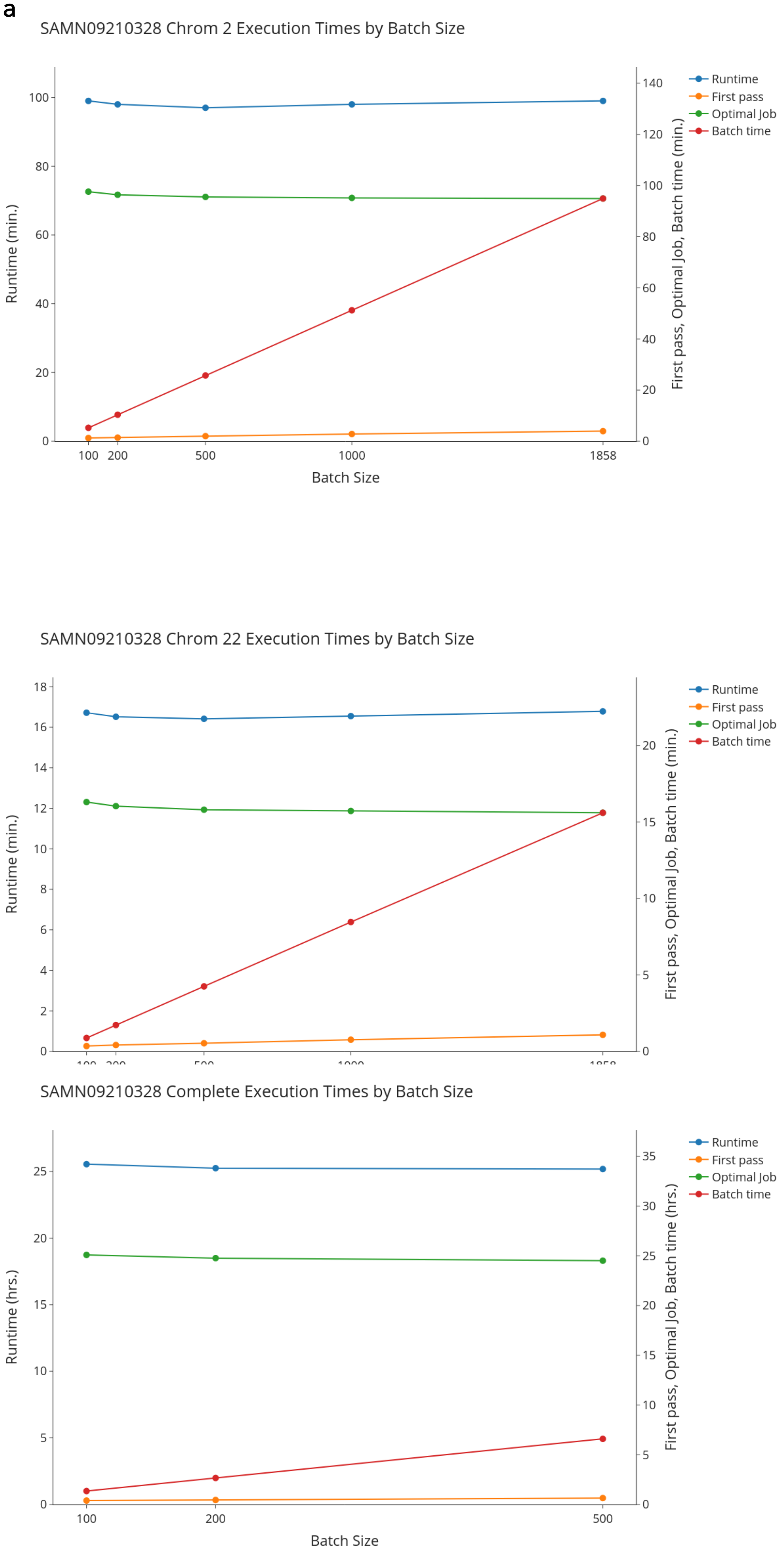

b

SAMN16086829 Chrom 2 Execution Times by Batch Size

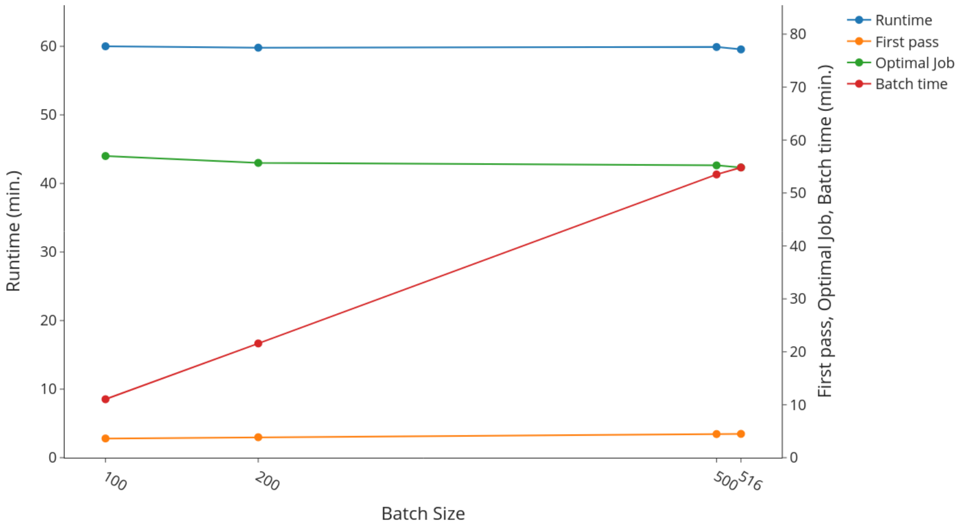

SAMN16086829 Chrom 22 Execution Times by Batch Size

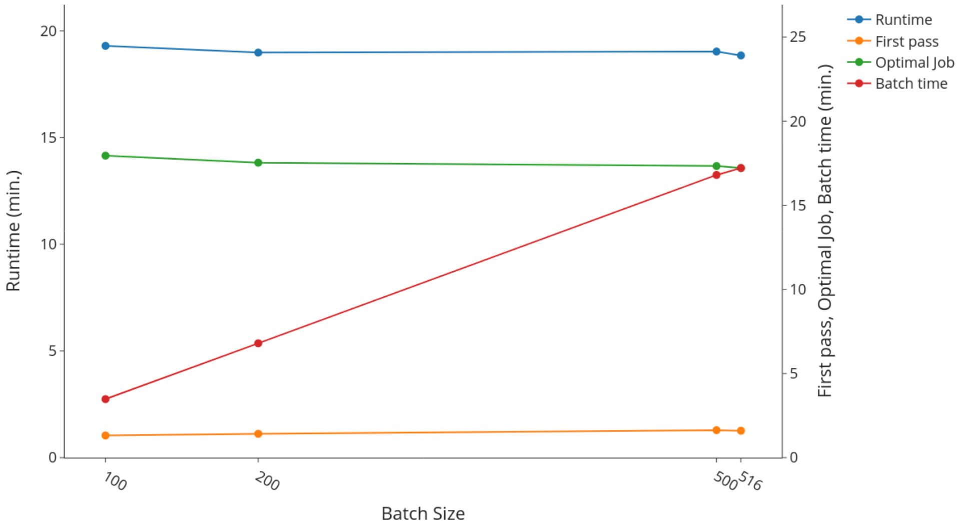

SAMN16086829 Complete Execution Times by Batch Size

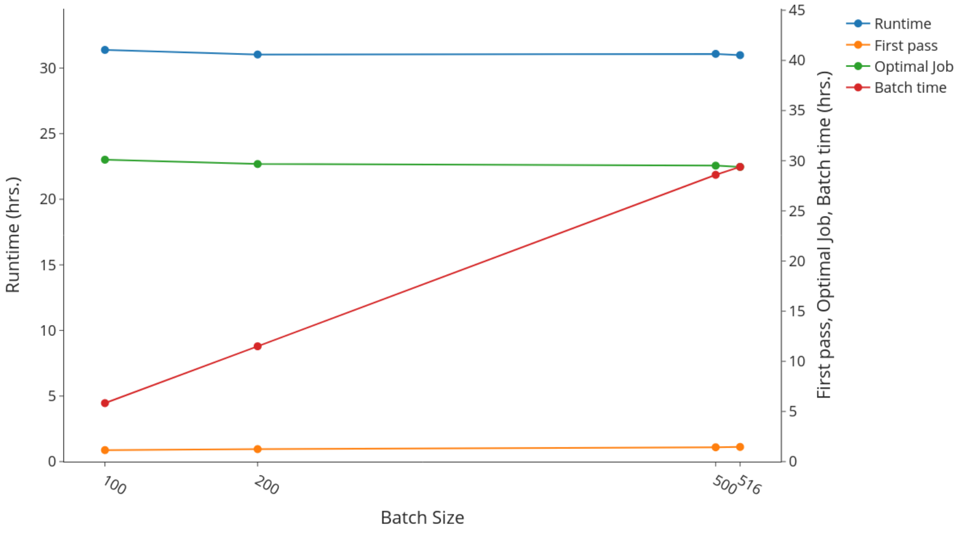

Figure_S7. SCExecute Memory Footprint by Batch Size

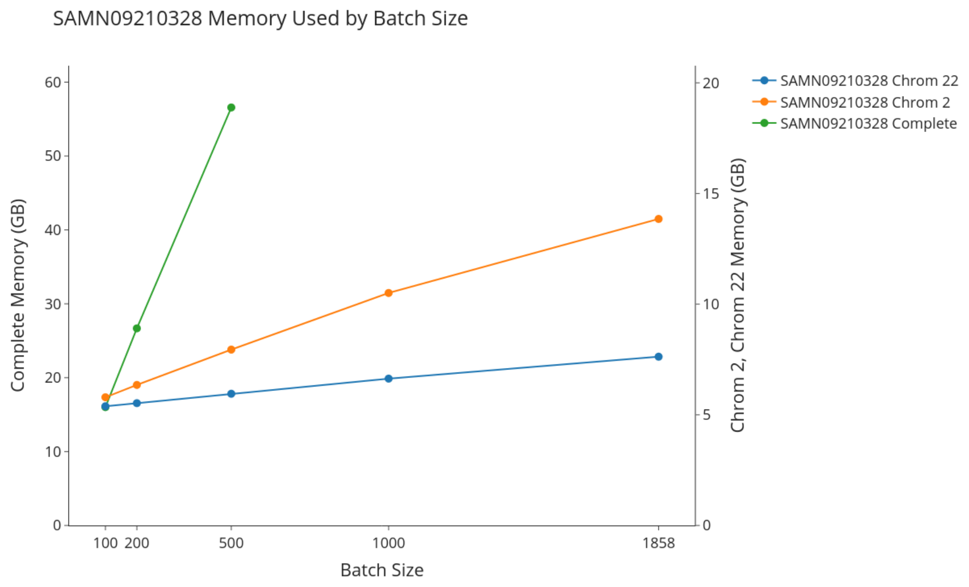

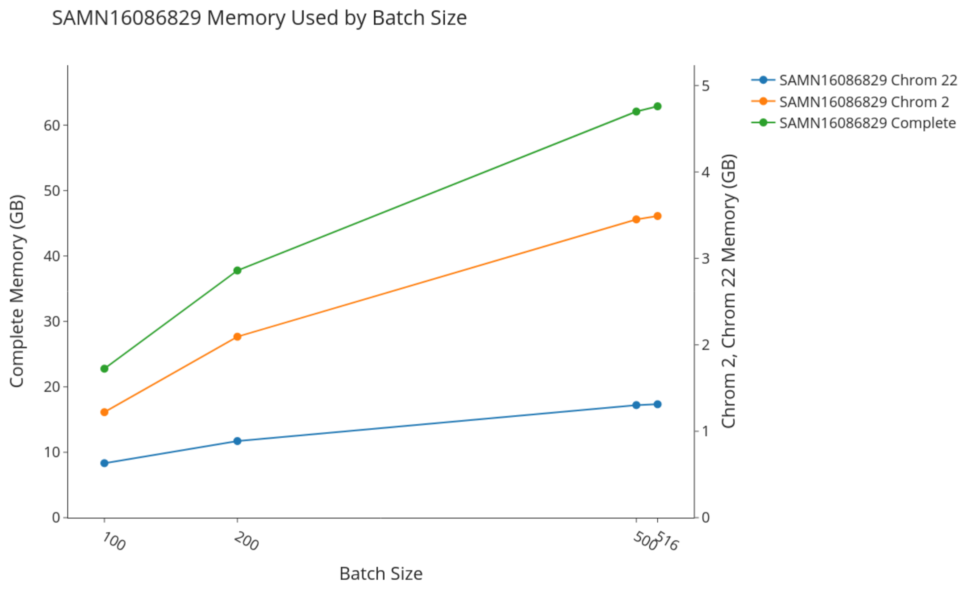

Discussion of SCExecute Performance

These figures demonstrate the performance of SCExecute and samtools-based workflows on different sized scRNA-Seq BAM files with different numbers of cellular barcodes and reads per barcode. We use restrictions to Chromosome 2 and Chromosome 22 to create smaller read sets to work with and vary the SCExecute Batch Size. We observe:

1. The samtools-based approach, which extracts each barcode's alignments one at a time independently and in parallel, requires 35%-66% more **Runtime** than the SCExecute approaches, even for small **Batch Size**.

2. The time to construct the first Batch Size cell-specific scBAM files (**First pass**) is approximately constant for all **Batch Size** values.

3. The **Optimal job** time indicates the time to execute the jobs on eight CPUs with minimal SCExecute overhead. It is not constant, because the average job execution time is less for larger **Batch Size** values, as discussed next.

4. The average time to execute jobs is affected by the **Batch Size**, because the machine has eight CPUs and eight workers are executing jobs. For the second and subsequent passes through the BAM file, the BAM File I/O thread is competing for CPU time with the workers.

5. The memory footprint increases with **Batch Size** to accommodate the reads of the cell-specific scBAM files for the current batch.

6. It is feasible, for some pooled scRNA-Seq BAM files, to use such a large **Batch Size** value that only the first pass is needed. The primary constraint here is the memory required to support this.

7. If the **Batch time** is larger than the **First pass** time (as shown in these examples), the workers will not finish execution on each batch's cell-specific scBAM files before the next pass through the scRNA-Seq file has completed, and the workers will not need to wait for more jobs after the first pass is done.

8. Once the **Batch time** is larger than the **First pass** time, the total running time of SCExecute is essentially constant, since the workers are never waiting for new scBAM files to be created after the **First pass**.
